# Translocation of intestinal bacteria as a cause of subcutaneous abscesses of the neck and head in American mink (*Neovison vison*) – a case report

**DOI:** 10.1186/s12917-020-02654-3

**Published:** 2020-11-10

**Authors:** Łukasz Wlazło, Wojciech Łopuszyński, Bożena Nowakowicz-Dębek, Mateusz Ossowski, Hanna Bis-Wencel

**Affiliations:** 1 Department of Animal Hygiene and Environmental Hazards, University of Life Sciences in Lublin, Akademicka 13, 20-950 Lublin, Poland; 2 Sub-Department of Pathomorphology and Forensic Veterinary Medicine, Department and Clinic of Animal Internal Diseases, University of Life Sciences in Lublin, Głęboka 30, 20-612 Lublin, Poland

**Keywords:** Mink (*Neovison vison*), Feed quality, Head and neck abscess, *Escherichia coli*

## Abstract

**Background:**

The problem of transmission of intestinal microorganisms to tissues occurs when intestinal epithelial cells do not adhere tightly (tight junction), which is caused by improper nutrition, usually associated with poor mucosal status. The impact on maintaining its proper condition in the case of animals also depends on the proper preparation and fragmentation of the ingredients of the feed. Intestinal microbiota disorders are increasingly indicated as one of the causes of many autoimmune, neurodevelopmental and metabolic diseases. However, there are no studies indicating damage to the intestinal barrier of animals resulting in the penetration of microorganisms from the gastrointestinal tract directly into the bloodstream which may result in the development of chronic inflammation.

**Case presentation:**

On a mink (*Neovison vison*) farm with a foundation stock of 4,000 females, abscesses were observed in the head, followed by progressive deaths. Antibiotic treatment with amoxicillin and clavulanic acid added to the animals’ feed was not successful. Macroscopic and microscopic changes indicated local suppurative inflammation of the skin and subcutaneous tissue with the presence of purulent fistulas. Microbiological analysis showed a significant increase in *Escherichia coli* in all samples taken from the abscesses. The results indicate the migration of intestinal bacteria through disturbance of the permeability of the intestinal barrier and their transfer to the blood. Symptoms were alleviated in all animals following changes in the feed components and in feed particle size.

**Conclusions:**

It is necessary to take into account the possibility of transmission of intestinal bacteria in the etiology of inflammatory diseases in animals. Conducting more research in this field will improve the understanding of the relationship between intestinal microbes and the health of the body as a whole.

## Background

The diet of animals is the most important factor affecting their productivity. In the case of carnivorous fur animals, this means obtaining optimal reproduction rates and skins with high quality parameters. The energy value of the feed rations should be adapted to the feeding period, and the feed should be preserved to protect it against the development of pathogenic microorganisms. Incorrect feeding of mink can lead to metabolic disorders, which are often imperceptible in the short production cycle of these animals. When selecting feed components, especially of animal origin, care should be taken about their microbiological status, freshness, high biological value and degree of homogenization [1, 2]. In addition to digestion and nutrient absorption, the digestive system also provides protection for the body. This is due to contact between gastrointestinal mucous membranes and factors ingested with food, which play an essential role in maintaining the body’s defence. Here antigens introduced with feed (e.g. bacteria) have the possibility of contact with immune cells, which enables the development of immune memory. The GALT (gut-associated lymphoid tissue) immune system plays an important role in local and systemic immunity. Within its structures antigens are presented to effector cells of the immune system. At these sites, antigens are captured by antigen-presenting cells (APCs), which by secreting appropriate cytokines and differentiating can lead to inflammation or antigen tolerance [3]. The intestinal ecosystem is constantly changing, but maintains a certain state of balance. Similarly, the composition of the intestinal microbiota changes depending on many environmental factors (e.g. pregnancy, lactation and diet), [4]. On the surface of mucous membranes there are beneficial intestinal commensal and probiotic bacteria [3, 5]. Bacteria that have an adverse effect on the body include Gram-negative anaerobes that produce endotoxins with pro-inflammatory properties. Species from the family *Enterobacteriaceae* (e.g. *Escherichia coli*) are the cause of intestinal infections when the body’s immune defences are weakened [6].

## Objective

The aim of the study is to present the problem of the occurrence of purulent skin lesions on the head and neck of farmed American mink (*Neovison vison*).

## Case presentation

The study was conducted in animals on a farm where the employees had observed abscesses on the heads of the mink, followed by progressive deaths. Information obtained from the breeder revealed that after the lesions appeared on the neck and head, the animals stopped feeding and gradually became weak, and after a few days deaths followed. At the same time, in a few individuals in which the abscess was opened spontaneously or mechanically due to scratching by the animals or veterinary intervention, the individual’s health improved and it was completely cured. No relapse was observed in these animals. In a few individuals the abscesses healed with no intervention and the animals’ health returned to normal. The abscesses appeared suddenly and frequently in the herd, and the employees noted from 30 to 50 new cases over the following week. The disease ultimately affected about 900 animals from a foundation stock of 4,000 females. Antibiotic treatment with amoxicillin and clavulanic acid added to the feed for 14 days was not successful in resolving the symptoms or preventing new cases. At the same time, the occurrence of new cases of the disease was observed to slow down, which indicated that it was bacterial.

To diagnose the problem, the breeder was asked to deliver several newly deceased animals to the Department, where post-mortem examination was performed. During the necropsy, organs were sampled for histological analysis. Following fixation in 10% neutral formalin, microscope slides were prepared of the samples by the paraffin technique and stained with haematoxylin and eosin (HE) and by special methods: periodic acid-Schiff (PAS), Gomori methenamine-silver (GMS) and Ziehl-Neelsen acid-fast (ZN) staining. Before the organ samples were taken, the lesions in the head of the animals were cut and samples were taken for microbiological testing. The material was plated on an agar medium with 5% sheep blood, MacConkey agar, and Sabouraud agar with chloramphenicol, and then incubated for 24 hours at 37 °C. The resulting colonies were identified using bioMerieux API biochemical assays.

**Fig. 1:**
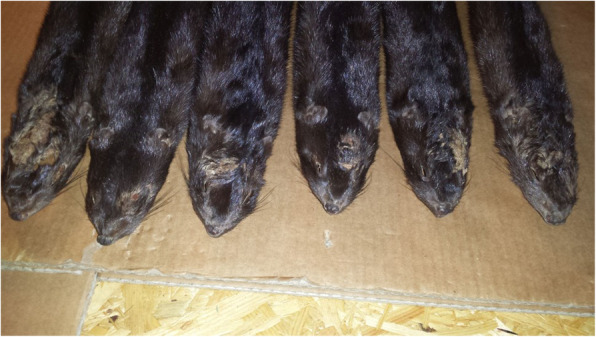
External gross lesions in necropsied minks. Focally extensive, hairless areas on the skin of scalp and neck covered with crusts

A complete necropsy with histological and microbiological examinations was performed on 6 randomly selected dead mink. External examination revealed dehydration and a decline in the animals’ body condition. Multifocal, variously shaped, coalescing, hairless areas of about 0.5 cm^2^ to about 12 cm^2^, covered with yellow-brown crusts, were observed on the scalp and dorsal surface of the neck (Fig. 1).

**Fig. 2:**
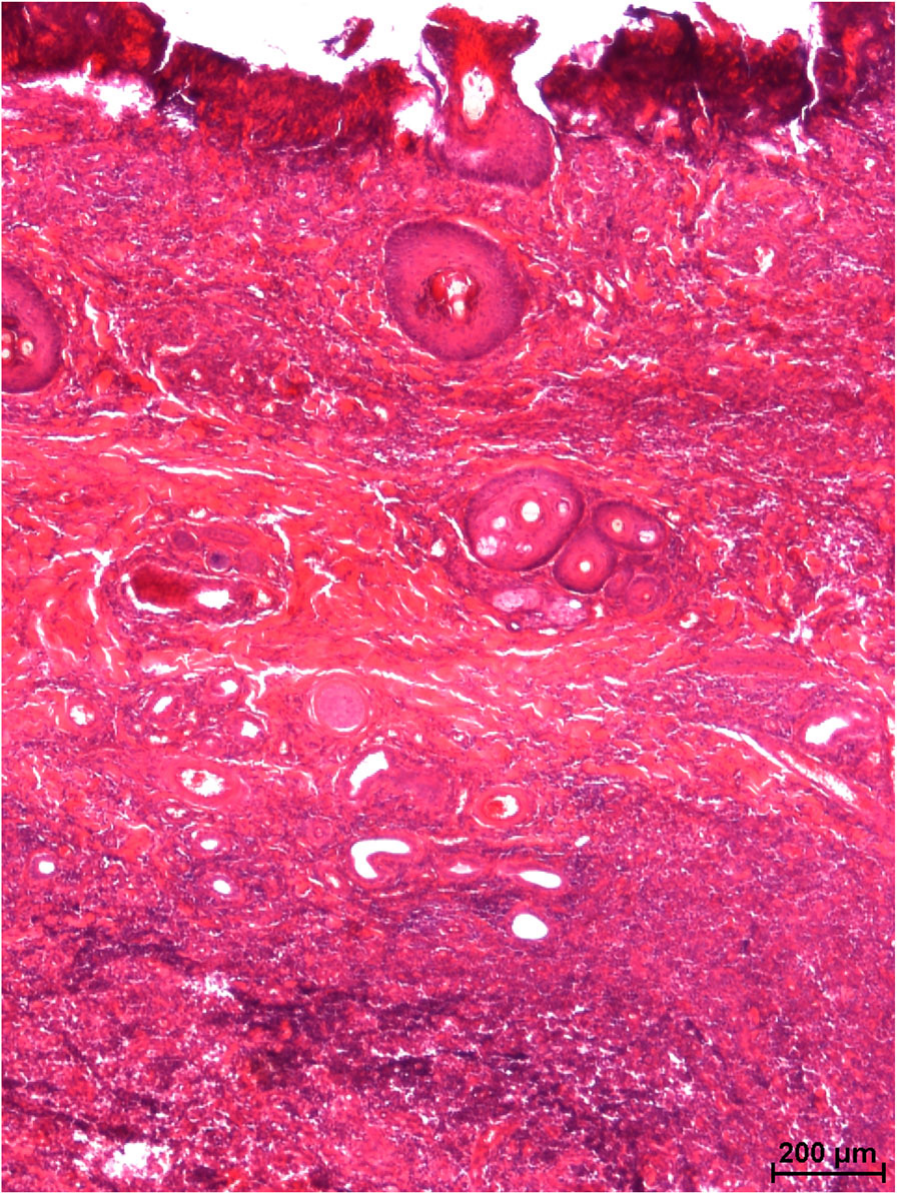
Severe diffuse pyogranulomatous inflammation extending from the ulcerated epidermis o the deep dermis and subcutis. HE. Bar = 200 µm

**Fig. 3:**
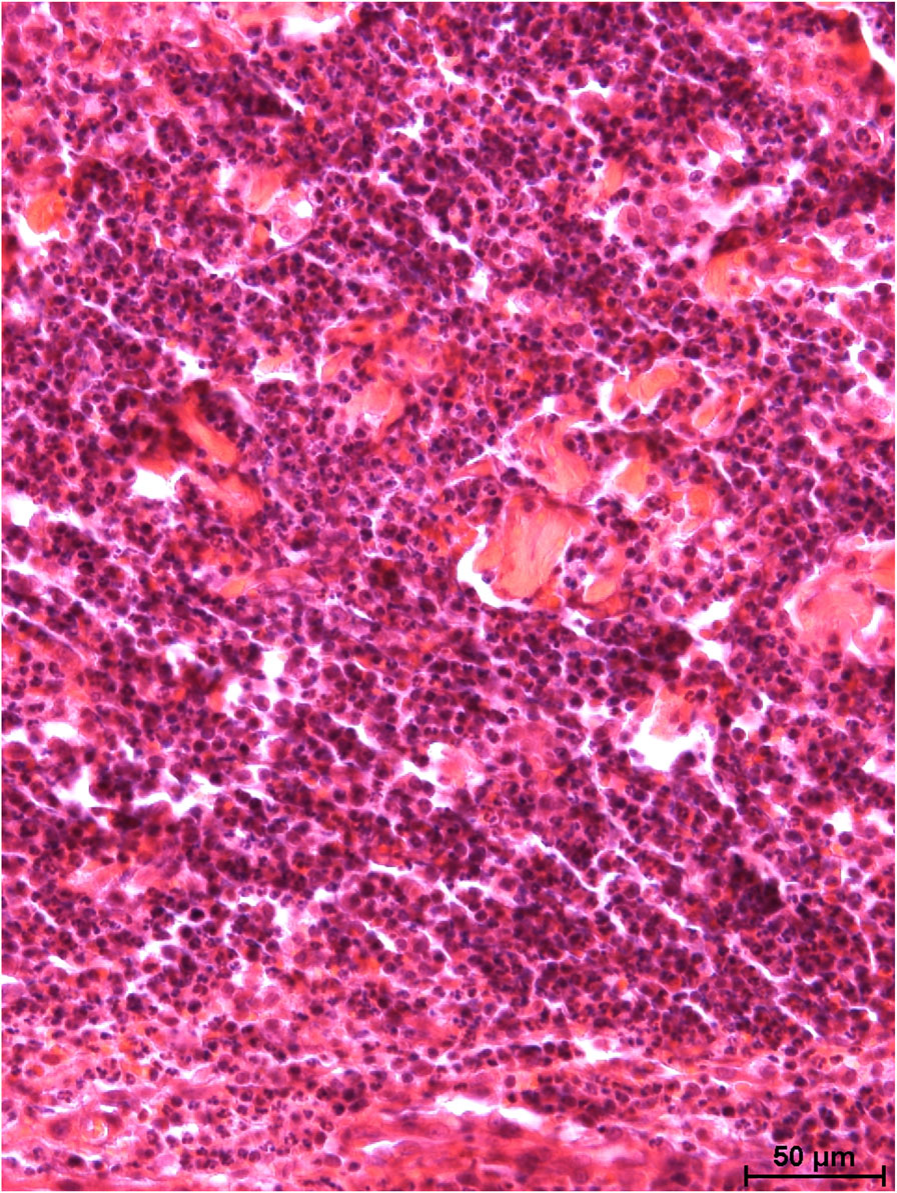
The inflammatory infiltrate compose of numerous viable and degenerate neutrophils, moderate numbers of macrophages, fewer lymphocytes and plasma cells admixed with cellular and karyorrhectic debris in the deep dermis. HE. Bar = 50 µm

Removal of the crust and compression of the skin revealed fistulas penetrating the skin and subcutaneous tissue, from which a purulent exudate oozed to the surface. The surrounding skin was significantly swollen. In 4 mink, moderate bilateral enlargement of the submandibular lymph nodes was evident. In addition, moderate splenomegaly, pulmonary oedema and congestion, and congestion of the liver and kidneys were found. No other macroscopic lesions were observed in internal organs. Microscopic examination of skin lesions revealed multifocal and coalescing nodular aggregates of numerous viable and degenerate neutrophils, moderate numbers of macrophages, and fewer lymphocytes and plasma cells admixed with cellular and karyorrhectic debris infiltrating the dermis and subcutis. Similar inflammatory cells were scattered between dermal collagen bundles and around the adnexal structures (Figs. 2 and 3). The overlying epithelium was focally ulcerated and covered with a serocellular crust. The additional staining methods (PAS, GMS and ZN) revealed no evidence of parasites, fungi, or mycobacteria. Gross and microscopic lesions were consistent with the diagnosis of focally extensive suppurative dermatitis and panniculitis with purulent fistulas.

Microscopic lesions in internal organs were limited to the liver. The periportal areas were multifocally infiltrated by low numbers of lymphocytes, plasma cells, fewer neutrophils, and macrophages, whereas multiple hepatocytes of centrilobular areas contained one to few clear cytoplasmic nucleus-displacing vacuoles (vacuolar change, lipid-type).

The microbiological analysis showed a very large increase in *Escherichia coli* in all samples taken from the abscess material. The results indicate the migration of intestinal bacteria through disturbance of the permeability of the intestinal barrier and their transfer to the blood. The interview with the breeder indicated that the feed ration was correctly balanced in terms of energy. Attention was drawn to the introduction of a feed component in the form of a 30% share of ground turkey bones, which coincided with the onset of disease among the animals and could have caused mechanical damage to the intestinal barrier.

## Discussion

Disturbances of intestinal permeability have been demonstrated to play a role in the pathogenesis not only of gastrointestinal diseases, but also of nervous, immune and reproductive disorders [7]. Translocation of gastrointestinal bacteria can be an important cause of the development of systemic infections, including opportunistic infections induced by phytophysiological bacterial flora. The causes of this phenomenon are most often found to be states of immunosuppression, imbalances of the microbiota causing excessive growth of Gram-negative intestinal bacilli, or mechanical disruption of the integrity of the intestinal mucosa [8]. Recent years have seen increased interest in the relationship between intestinal microbiota and nervous system function or animal health. Communication along the gut-brain axis indicates that the composition of the gut microbiota determines normal brain activity. A well-functioning intestinal barrier restricts the penetration of pathogenic microorganisms into the blood. Dysbiosis, i.e. abnormal composition of the microbiota, disturbs the functioning of this barrier (leaky gut syndrome). This leads to increased migration of antigens and intestinal bacteria into the blood. This triggers an immune response, and inflammatory factors that accumulate contribute to the development of disorders [9–11]. The intestine is a hormonally active organ, secreting mucus and class A immunoglobulins, which are responsible for protection against harmful elements of feed, bacteria and their toxins. There is abundant lymphoid tissue (GALT) in the intestine. In the development of the inflammatory process, an important role is attributed to immunological damage to the intestinal mucosa and to pro- and anti-inflammatory cytokines characteristic of the cellular and humoral immune response. 16S rDNA sequence analysis has been used to detect the presence of invasive *E. coli* strains in the colonic mucosa of dogs with granulomatous colitis [12–14].

To alleviate the symptoms in all animals and prevent remission of the disease, it was recommended that the ground turkey bones should be eliminated from the diet, as they were believed to have caused the damage to the intestinal mucosa. For economic reasons and in order to maintain the proper structure and energy value of the feed, the breeder chose to replace the turkey bones with poultry breast bones, but ground much more finely. This resulted in a significant reduction in the incidence of new cases of disease. Complete remission of the lesions was observed in the animals a few weeks after the formula and means of preparing the feed had been changed.

## Conclusions

Maintaining the proper state of intestinal epithelium depends on many factors, including the proper preparation of animal feed. Intestinal microorganisms that can enter the blood and tissue of animals through the intestinal epithelium should be considered in the etiological differentiation of disease lesions. A thorough understanding of bacterial translocation mechanisms will allow effective therapy of the source of infection without re-emission of inflammatory processes.

## Data Availability

Not applicable.

## Abbreviations

HE: Haematoxylin and eosin staining
PAS: Periodic acid-Schiff staining.
GMS: Gomori methenamine-silver staining.
ZN: Ziehl-Neelsen acid-fast staining.
GALT: Gut-associated lymphoid tissue.
